# Diagnostic classification of children and adolescents with high-functioning autism spectrum disorder based on brain functional network characteristics of the dense individualized and common connectivity-based cortical landmark model

**DOI:** 10.3389/fpsyt.2026.1892660

**Published:** 2026-08-18

**Authors:** Yonglu Wang, Jingjing Ma, Zhengwang Xia, Cheng Song, Yang Zong, Shasha Xiao, Xiaoyan Ke, Hui Fang, Yun Li

**Affiliations:** 1 Child Mental Health Research Center, the Affiliated Brain Hospital of Nanjing Medical University, Nanjing, China; 2 School of Computer Information and Engineering, Changzhou Institute of Technology, Changzhou, China; 3 The Affiliated Brain Hospital of Nanjing Medical University, Nanjing, China; 4 Faculty of Sports & Exercise Science, University of Malaya, Kuala Lumpur, Malaysia

**Keywords:** connectome signatures, dense individualized and common connectivity-based cortical landmarks (DICCCOL), diffusion tensor imaging (DTI), high-functioning autism spectrum disorder (HF-ASD), magnetic resonance imaging (MRI)

## Abstract

**Objective:**

To investigate whether structure-informed functional connectivity patterns derived from the Dense Individualized and Common Connectivity-based Cortical Landmarks (DICCCOL) framework can distinguish children and adolescents with high-functioning autism spectrum disorder (HF-ASD) from typically developing (TD) controls, and to explore the clinical relevance of the identified connectivity features.

**Methods:**

Multimodal magnetic resonance imaging data, including diffusion tensor imaging (DTI) and resting-state functional MRI (rs-fMRI), were acquired from 37 participants with HF-ASD and 33 TD controls. A total of 358 DICCCOL landmarks were localized in each participant’s individual brain space based on DTI-derived white matter connectivity patterns. rs-fMRI data were aligned to the corresponding DTI space, and whole-brain functional connectivity was calculated among DICCCOL landmarks. Classification was performed using a linear support vector machine within a fully nested leave-one-out cross-validation framework. All supervised procedures, including FDR-corrected group comparisons, correlation-based feature selection, feature standardization, and hyperparameter optimization, were conducted exclusively within the training data of each cross-validation iteration. Stable discriminative functional connections were further characterized according to their functional network affiliations, and exploratory associations with clinical measures were examined.

**Results:**

The DICCCOL-based functional connectivity model achieved an out-of-fold classification accuracy of 84.29%, with a sensitivity of 83.78%, a specificity of 84.85%, and an area under the receiver operating characteristic curve of 0.832. The stable discriminative functional connections included both increased and decreased connectivity in the HF-ASD group and involved both intra-network and inter-network interactions. These connections were primarily distributed across cognitive-cognitive, cognitive-affective, and affective-affective systems. In addition, several stable functional connections showed significant negative associations with clinical measures, including ADI-R total scores, ADI-R Social Interaction scores, and GEM-PR scores, suggesting potential links between altered connectivity patterns and individual differences in autism-related symptom burden, social functioning, and empathic ability.

**Conclusions:**

Structure-informed functional connectivity features based on individualized DICCCOL landmarks demonstrated good discriminative potential for identifying HF-ASD in the present sample. The identified connectivity patterns may reflect altered functional integration across cognitive and affective systems and may be related to clinical heterogeneity in ASD. These findings should be considered preliminary, and the identified patterns should be regarded as candidate neuroimaging signatures rather than established diagnostic biomarkers. Validation in larger, longitudinal, independent, and multi-center cohorts is warranted.

## 1 Introduction

Autism spectrum disorder (ASD) is a neurodevelopmental condition characterized by persistent impairments in social communication and social interaction, together with restricted and repetitive patterns of behavior or interests. Increasing evidence suggests that ASD cannot be fully explained by abnormalities in isolated brain regions, but is more appropriately understood as a disorder involving altered large-scale brain network organization and inefficient communication among distributed neural systems. Resting-state functional magnetic resonance imaging (rs-fMRI) has therefore been widely applied to investigate functional dysconnectivity in ASD, including alterations in within-network and between-network interactions relevant to social cognition, executive control, self-referential processing, and affective regulation (1). Recent multi-site rs-fMRI studies have further examined ASD-related topological alterations in functional brain networks using graph-theoretical approaches (2). In parallel, machine-learning studies using independent component analysis (ICA)-derived spatial-temporal features or spatially constrained ICA networks have demonstrated the potential of rs-fMRI-based functional network measures for ASD classification across multiple sites (3, 4). These advances support the potential value of network-level neuroimaging features for characterizing ASD; however, differences in sample composition, acquisition protocols, node definitions, feature extraction strategies, and validation procedures continue to limit direct comparison across studies and may affect the reproducibility and generalizability of reported classification performance.

A major methodological challenge in functional connectome research is the definition of network nodes that can be reliably compared across individuals. Conventional functional connectivity studies commonly rely on atlas-based parcellation schemes or relatively large anatomically defined regions of interest (ROIs) (5, 6). Although such approaches are widely used, they generally depend on inter-individual image registration to a standard space and may not adequately capture individual variability in cortical organization and white matter connectivity. In children and adolescents with ASD, in whom neurodevelopmental heterogeneity is substantial, imperfect inter-subject correspondence may further obscure biologically meaningful network differences. Therefore, establishing individualized yet cross-subject comparable network nodes is important for improving the precision of functional connectome analyses and for identifying reproducible neuroimaging signatures.

The Dense Individualized and Common Connectivity-based Cortical Landmark (DICCCOL) framework provides a structure-informed approach to this problem. DICCCOL landmarks are defined in each individual brain on the basis of consistent white matter fiber connection patterns and are optimized to preserve corresponding structural connectivity profiles across individuals. Rather than relying solely on the spatial correspondence imposed by a standard atlas, DICCCOL enables the construction of individualized cortical landmarks with cross-subject structural correspondence (7–9). This property makes DICCCOL particularly suitable for integrating diffusion tensor imaging (DTI) and rs-fMRI data to investigate large-scale structural-functionally informed connectomes (10, 11). More broadly, recent methodological developments have highlighted the value of examining topological organization and hub-related properties in brain networks (12, 13). Although these studies were not specifically focused on ASD, they emphasize that network topology, nodal importance, and distributed connectivity patterns may provide complementary information beyond pairwise functional connections alone. In this context, DICCCOL-based functional connectivity analysis offers a complementary strategy by prioritizing individualized, structurally grounded cortical correspondence before network-level feature extraction and classification (14–16).

Accordingly, the present study aimed to investigate DICCCOL-based functional connectivity signatures in children and adolescents with high-functioning ASD (HF-ASD) (17). Multimodal MRI data, including DTI and rs-fMRI, were acquired from HF-ASD participants and typically developing (TD) controls (18). First, 358 DICCCOL landmarks were localized in each participant’s individual brain space based on DTI-derived white matter connectivity patterns. Functional connectivity among these landmarks was then calculated using rs-fMRI time series to construct an individualized whole-brain functional connectome. Next, a two-stage feature-selection strategy and a fully nested support vector machine classification framework were used to identify stable discriminative functional connectivity features and evaluate their out-of-fold classification performance while minimizing information leakage and overfitting (19). Finally, the functional roles of the identified connections were characterized, and their relationships with clinical measures were explored. By integrating individualized structural correspondence, whole-brain functional connectivity analysis, and rigorously validated machine learning, this study sought to provide a structure-informed perspective on candidate functional connectome signatures associated with HF-ASD (20).

## 2 Materials and methods

### 2.1 Ethics statement

This study was approved by the Medical Ethics Committee of the Brain Hospital Affiliated to Nanjing Medical University (Ethics Approval No. 2022-KY022-01). Prior to commencing the experimental procedures, the parents or legal guardians of the participants were informed of the purpose of the study, as well as detailed procedures, and were requested to sign the informed consent form (children > 8 years of age with normal cognitive capacity were also required to independently provide their signatures for confirmation).

### 2.2 Participants (9)

All participants were recruited from the Nanjing Medical University Autism Cohort (this cohort is dedicated to disease-specific research on autism, prospectively collecting rsfMRI, electroencephalography, as well as urine and stool samples from children with ASD). The study included six sub-cohorts: sibling-matched, twin, early intervention, very early intervention, child-adolescent, and typically developing groups, with an enrollment period from July 2023 to July 2025.

The HF-ASD Group: Participants first completed clinical psychological assessments, and were then diagnosed by two senior child psychiatrists according to the relevant diagnostic criteria of the Diagnostic and Statistical Manual of Mental Disorders, Fifth Edition (DSM-5) (21). Individuals with inconsistent diagnoses were excluded from the study. The inclusion criteria were the following: (1) patients met the DSM-5 diagnostic criteria for autism spectrum disorder (ASD); (2) participants were 6–16 years of age, (3) participants had an intelligence quotient (IQ) of 80 or above, (4) all participants were right-handed; and (5) all participants obtained guardian consent to participate in the study. Exclusion criteria were the following: (1) a history of traumatic brain injury; (2) suffering from neurological disorders or severe somatic diseases; and (3) diagnosed with specific mental disorders such as depression, mania, attention deficit hyperactivity disorder, or learning disabilities. A total of 37 children and adolescents with HF-ASD were enrolled, comprising 32 males and five females, with a mean age of (9.15 ± 2.22) years.

Typically developing (TD) control group: During the same period, 39 TD children matched with the patient group in age, sex, and IQ were enrolled as controls. Inclusion criteria were the following: (1) normal physical and cognitive development, and (2) guardian consent was obtained. Exclusion criteria were the following: (1) a history of traumatic brain injury, (2) a history of neurological disorders or severe somatic diseases, and (3) diagnosed with other specific mental disorders. A total of 33 subjects were enrolled, comprising 24 males and nine females, with a mean age of (10.05 ± 3.20) years.

### 2.3 Clinical psychological assessment

All enrolled participants underwent detailed assessments and data documentations conducted by trained professionals in psychology or psychiatry at the Child Psychology Research Center of Nanjing Medical University Affiliated Brain Hospital. The documentation included: ① General information survey: A self-developed questionnaire was used to investigate the overall demographic data, birth history, past medical history, and family history of all participants. ② Clinical symptom assessment: The Autism Diagnostic Observation Schedule, Second Edition (ADOS-2) (22) and the Autism Diagnostic Interview-Revised (ADI-R) (23) were used for clinical diagnostic evaluation; the Wechsler Intelligence Scale for Children, Fourth Edition, Chinese Revision was used to assess the IQ of all participants (24); and the Griffith Empathy Measure Parent Ratings (GEM-PR) (25) was used to evaluate the cognitive and affective characteristics of all participants.

### 2.4 Image data acquisition

A 3.0T superconducting MRI system manufactured by Siemens (Munich, Germany) was used with a birdcage head quadrature coil, with the head stabilized with foam padding. Each participant first underwent axial T2-weighted imaging (T2WI) for anatomical localization and to exclude individuals with visible neurological organic diseases (26). A spin-echo sequence was used to acquire sagittal T1-weighted images of the head. The scanning parameters were as follows: repetition time (TR) = 2, 530 ms, echo time (TE) = 3.34 ms, slice thickness = 1.33 mm, flip angle = 7°, inversion time (TI) = 1, 100 ms, field of view (FOV) = 256 × 256 mm², and matrix = 256 × 192, total acquisition time = 8 min and 7 s. The scanning slices were oriented parallel to the line connecting the genu and splenium of the corpus callosum, covering from the top of the head to the inferior part of the cerebellum, with a total of 128 slices acquired. The scanning parameters for rsfMRI data acquisition were as follows: gradient echo pulse sequence - echo planar imaging (EPI) sequence, repetition time (TR) = 2, 000 ms, echo time (TE) = 30 ms, acquisition matrix = 64 × 64, flip angle = 90°, FOV = 240 × 240 mm², slice thickness = 4 mm, number of slices = 30, slice gap = 0 mm, and total acquisition time = 4 min 44 s. The scanning parameters for DTI data acquisition were as follows: diffusion sensitivity coefficient (b-value) = 1, 000 s/mm², 30 diffusion-sensitive gradient directions, acquisition matrix = 128 × 128, FOV = 230 mm × 230 mm, slice thickness = 1.33 mm, TR = 9, 000 ms, and TE = 3.34 ms, total acquisition time = 5 min 8 s, with 60 slices acquired per scan (27).

#### 2.4.1 Data preprocessing

**Figure 1:**
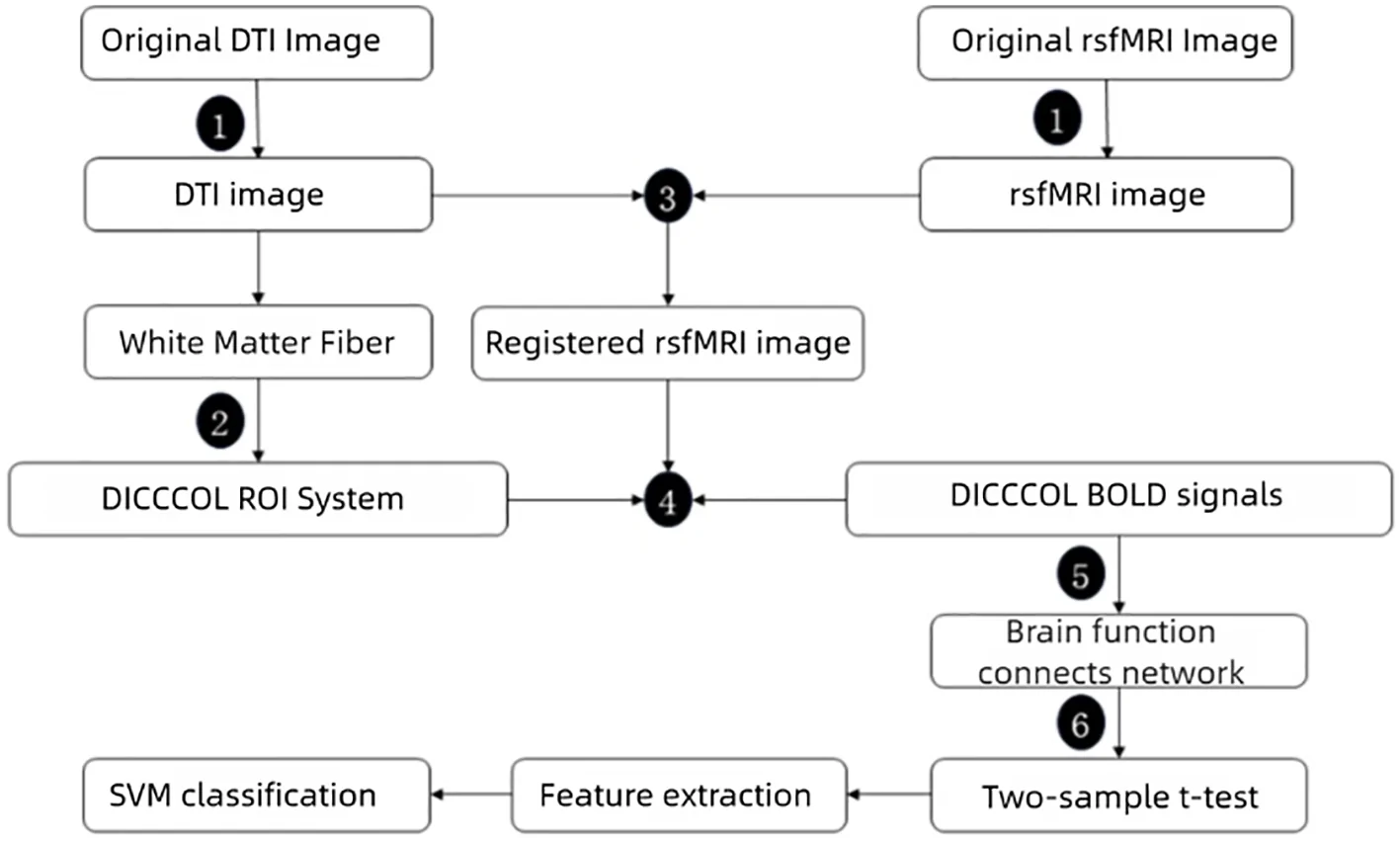
Basic flowchart of the dense individualized and common connectivity-based cortical landmarks model-based activation detection. DTI, Diffusion Tensor Imaging; rsfMRI, Resting-State Functional Magnetic Resonance Imaging; ROI, Region of Interest; DICCCOL, Dense Individualized and Common Connectivity-based Cortical Landmark; SVM, Support Vector Machine; BOLD, Blood Oxygen Level Dependent.

The data preprocessing steps included skull stripping, motion correction, spatial smoothing, temporal pre-whitening, slice timing correction, global drift removal, and bandpass filtering (0.01 Hz~0.1 Hz) (28). The preprocessing steps for DTI data included skull stripping, motion correction, and eddy current correction. Following preprocessing, fiber tracts, gray matter (GM), and white matter (WM) tissues were segmented based on the DTI data, and GM/WM cortical surfaces were reconstructed (29). Fiber tracking was performed using MEDINRIA (http://www-sop.inria.fr/asclepios/software/MedINRIA/). Direct segmentation of brain tissue was obtained from DTI data (30). Based on the WM tissue map, the cortical surface was reconstructed using the Marching Cubes algorithm (31). The reconstructed surface, comprising approximately 40, 000 vertices, was used as the standard space for predicting DICCCOL (15, 32), as shown in **Figure 1** (1).

The basic workflow of the DICCCOL-based activation detection data analysis method is summarized in **Figure 1**. (1) Diffusion tensor imaging (DTI) and rest state functional magnetic resonance imaging data preprocessing. (2) DICCCOL prediction: DTI images were registered to the same fMRI image space using the FSL FLIRT tool (http://www.fmrib.ox.ac.uk/fsl/). White matter fibers in the DTI data were tracked using MedINRIA (http://www-sop.inria.fr/asclepios/software/MedINRIA/). Then, we localized and optimized 358 DICCCOL nodes in the individual brain space using the method proposed by Da-Jiang Zhu et al (15). (3) Image registration and (4) BOLD signal extraction: Based on the DTI data and task-based functional image data in the same space, we extracted the time series corresponding to each DICCCOL node. We then used the conventional GLM method to estimate the magnitude and extent of stimulus-evoked activation responses within each individual’s space. Leveraging the inherent structural and functional consistency of the 358 DICCCOL nodes, we used a group-level mixed-effects statistical model to compute the statistical effects of each DICCCOL node, thereby obtaining a group-consistent distribution map of activated DICCCOL nodes. (5) Brain functional connectivity network construction and (6) brain functional connectivity network analysis.

#### 2.4.2 DICCCOL prediction

We used a novel data-driven strategy and identified 358 consistent ROIs with corresponding relationships across six independent datasets comprising over 200 healthy brains. Each identified ROI was optimized to achieve maximum group consistency in DTI-based fiber shape patterns. This set of 358 ROIs was termed the DICCCOL model (https://www.nitrc.org/projects/dicccol) (15). Briefly, through a group-by-group optimization process using the trace map model (33), 358 landmarks were stabilized from over 2, 000 candidates, each exhibiting intrinsic consistency across different individuals. During this process, only WM fiber bundles that precisely penetrated or passed through a small neighborhood (2 mm radius) of the target location were considered. By adopting the prediction framework, we effectively transferred the 358 DICCCOLs to a new brain. The prediction process resembled the DICCCOL optimization procedure; the structural profile consistency between the new brain and the DICCCOL model was maximized, as shown in Equation 1:

(1) $$\text{E}(S_{M},S_{N})=\sum |D_{MN}|$$

**Figure 2:**
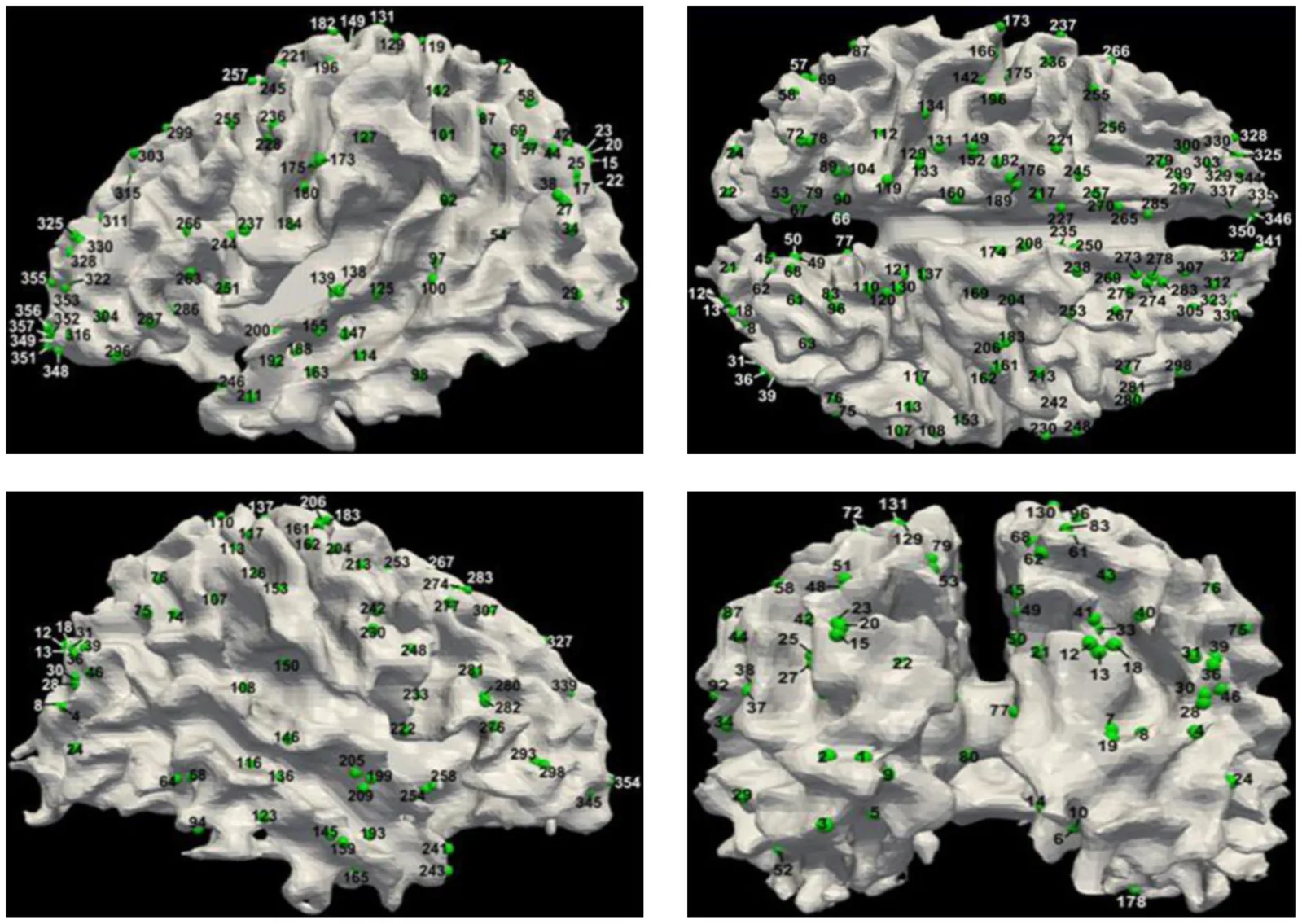
An example of 358 dense individualized and common connectivity-based cortical landmark models distributed on the cortical surface.

Where SM represents the DICCCOL model, SN denotes the new brain to be predicted, and DMN is defined as the trace map distance (34). The trace map distance represented the similarity of connectivity patterns between two independent WM fiber bundles (35). An example of 358 DICCCOLs distributed on the cortical surface is shown in **Figure 2**. For further details on algorithms and results, please refer to the website (https://www.nitrc.org/projects/dicccol_0_1), as illustrated in **Figure 1** (5).

#### 2.4.3 Constructing a FC matrix

Functional Connectivity (FC) construction: Because both fMRI and DTI sequences were EPI (echo-planar imaging) sequences, their distortions tended to be similar. The mismatch between fMRI and DTI images was significantly smaller than those between rsfMRI and T1-weighted images (36). Because DICCCOLs were defined on the cortical surface (GM) (20) we could significantly reduce variance and noise [such as from WM and cerebral spinal fluid (CSF) that were unlikely to reflect neuronal activity when extracting rsfMRI BOLD signals based on DICCCOLs (37). Therefore, for each retained DICCCOL, its rsfMRI time series was obtained by averaging within a small neighborhood (three surface mesh rings, approximately 3 mm in radius). We evaluated the FCs between each pair of DICCCOLs using Pearson’s correlation coefficient and constructed an M × M symmetric matrix for subsequent analysis. Here, M equals the number of retained DICCCOLs, as shown in **Figure 1** (8).

#### 2.4.4 Feature selection

Given the high dimensionality of the whole-brain FC matrix, a two-stage supervised feature selection procedure was implemented to reduce dimensionality and identify informative FC features. Crucially, to prevent information leakage, all supervised feature selection steps were performed exclusively within the training data of each cross-validation fold. No group-level statistical testing, feature selection, feature scaling, or classifier parameter optimization was conducted on the entire dataset prior to cross-validation (38, 39).

For each training set, a two-sample t-test was applied to every FC feature to compare the HF-ASD and TD groups. Multiple comparisons were corrected using the false discovery rate (FDR), and features with *q* < 0.05 were retained for the next stage. This initial screening was designed to eliminate FCs with weak univariate discriminative information while significantly reducing the dimensionality of the feature space.

Given that the FDR-corrected t-test evaluates each FC feature independently and does not account for redundancy among the retained features, correlation-based feature selection (CFS) was subsequently applied to the features that passed the initial screening. CFS evaluates a candidate feature subset by jointly considering each feature’s association with the class label and the intercorrelations among features (40, 41). For a feature subset *S* consisting of *k* features, the subset merit is computed as:


$$Merit_{s}=\frac{kr_{cf}}{\sqrt{k+k(k-1)r_{ff}}}$$

where *r_cf_* denotes the mean feature-class correlation, and *r_ff_* denotes the mean feature-feature intercorrelation. A subset with stronger feature-class associations and lower inter-feature redundancy is preferred.

#### 2.4.5 Performance evaluation

A fully nested cross-validation framework was employed to evaluate the generalizability of the classification model while preventing information leakage. The outer loop used leave-one-out cross-validation (LOOCV), in which one participant was held out as an independent test sample in each iteration, and the remaining participants formed the outer training set.

Within each outer training set, an inner stratified 5-fold cross-validation procedure was conducted to optimize the regularization parameter *C* of the linear support vector machine (SVM). For each inner-loop split, all data-dependent steps were repeated using only the inner training samples. Specifically, this involved FDR-corrected two-sample t-tests, followed by CFS-based feature selection. The selected FC features were then standardized using the mean and standard deviation calculated solely from the inner training data. The same feature-selection indices and scaling parameters were subsequently applied to the corresponding inner validation set. A linear SVM model was trained for each candidate value of *C* from a predefined grid (e.g., 0.00001, 0.0001, …, 0.1), and the optimal parameter was selected based on the mean balanced accuracy across the inner folds.

After identifying the optimal hyperparameter, the entire feature selection procedure was repeated on the complete outer training set. Specifically, FDR-corrected t-tests and CFS were performed exclusively on the outer training data. The selected features were then standardized using parameters derived from the same training set. A linear SVM classifier was subsequently trained on the outer training data and applied to the held-out test participant.

The aforementioned procedure was repeated until each participant had served as the outer test sample once. Performance metrics-accuracy, sensitivity and specificity-were computed from the aggregated out-of-fold predictions, from which a confusion matrix and an ROC curve was also generated (42–44).

To determine whether the observed performance exceeded chance levels, we employed a nonparametric permutation test. Specifically, we conducted 5000 random permutations of the diagnostic labels while keeping the functional connectivity (FC) feature matrix fixed. For each permuted dataset, we reran the entire nested cross-validation pipeline—including inner-loop parameter optimization, FDR-based feature screening, CFS, feature scaling, SVM training, and outer-loop testing—following the same protocol used for the true labels (**Supplementary Figure 1**).

#### 2.4.6 Identify FCs with high differentiation

To some extent, aberrant functional alterations in HF-ASD patients can be represented by FCs with high discriminative power (38). However, reducing the number of features (e.g., pairwise correlations) is an effective and sometimes unavoidable approach to accelerate computation and mitigate noise, including positively correlated errors when only pairwise nodes are considered (39). Thus, our strategy involved using a two-stage supervised feature selection procedure, retaining only the most distinctive and descriptive features to differentiate between HF-ASD patients and healthy controls.

Because we only had two categories (HF-ASD patients and normal controls), we used a straightforward false discovery rate (FDR)-corrected *t*-test *(*40) in the first stage to eliminate connections that showed no significant difference between the two disease/control groups. However, the *t*-test evaluated these connectivity features individually, meaning it did not account for correlations between features, and thus failed to capture redundancy among the retained features. To address this issue, we used the correlation-based feature selection (CFS) algorithm as the second stage of feature selection. The core idea of CFS was to evaluate the merits of feature subsets through a heuristic process, which considered both the predictive power of individual features for class labels, and the degree of intercorrelation among them (40).Unlike the first-stage *t*-test, CFS simultaneously computed feature-class and feature-feature intercorrelations (41). Given a feature subset S with k features, the Merit was defined as follows:


$$\frac{\text{k}\bar{\text{corre}(\text{c},\text{ f})}}{{\sqrt{\text{k}+(\text{k}-1)\bar{\text{corre}(\text{f},\text{ f})}}}^{2}}$$

where $\bar{\text{corre}(\text{c},\text{ f})}$ and $\;\bar{\text{corre}(\text{f},\text{ f})}$ represent the mean feature-class correlation and the mean feature-feature intercorrelation, respectively.

#### 2.4.7 Support vector machine classification and cross-validation

After obtaining the most prevalent and discriminative connectivity features through this two-stage feature selection process, a support vector machine classifier with a linear kernel (42) was used to address the classification problem (43). Given the limited number of participants in the dataset, we adopted the commonly used Leave-One-Out Cross-Validation strategy to evaluate the sensitivity (proportion of correctly predicted patients) and specificity (proportion of correctly predicted healthy controls) of our selected features (44).

## 3 Results

### 3.1 Demographic characteristics of the two participant groups

There was no significant difference in sex (χ² = 2.064, p = 0.231), age (*t* = -0.008, p = 0.993), or IQ (t = -0.096, p = 0.924) between the two groups of children and adolescents. The mean scores across the three ADI-R domains, Reciprocal Social Interaction, Communication, and Restricted, Repetitive Behaviors, all met the diagnostic thresholds (**Table 1**).

**Table 1:** Demographic and clinical characteristics of the HF-ASD and TD groups.

Variables	HF-ASD	TD	t/χ2
Sex (Male/Female)	32/5	24/9	2.064
Age	9.8 ± 2.705	9.806 ± 3.183	-0.008
FIQ	106.554 ± 22.772	107.03 ± 18.091	-0.096
ADI-R total	27.135 ± 12.869		
ADI-R Social Interaction	13.838 ± 7.274	–	–
ADI-R Verbal	9.243 ± 4.979	–	–
ADI-R Stereotypy	4.054 ± 2.656	–	–
ADOS-2			
ADOS-2 Communication	4.027 ± 2.179	–	–
ADOS-2 Social Interaction	7.351 ± 2.955	–	–
ADOS-2 Stereotypy	2.811 ± 2.158	–	–
CARS	29.865 ± 4.785		
GEM-PR Total Score	11.541 ± 18.668	28.303 ± 20.283	-3.6*
GEM-PR Cognitive Subscale	4.189 ± 10.482	22.697 ± 19.116	-5.095**
GEM-PR Affective Subscale	11.649 ± 17.831	17.212 ± 13.788	-1.447

FIQ, full scale of intelligence quotient; ADI-R, Autism Diagnostic Interview-Revised; ADOS, Autism Diagnostic Observation Schedule; HF-ASD, high functioning-autism spectrum disorder; CARS, childhood autism rating scale; GEM-PR, Griffith Empathy Measure-Parent Rating. ^*^p < 0.05; ^**^p < 0.001.

### 3.2 Classification results based on FCs

**Figure 3:**
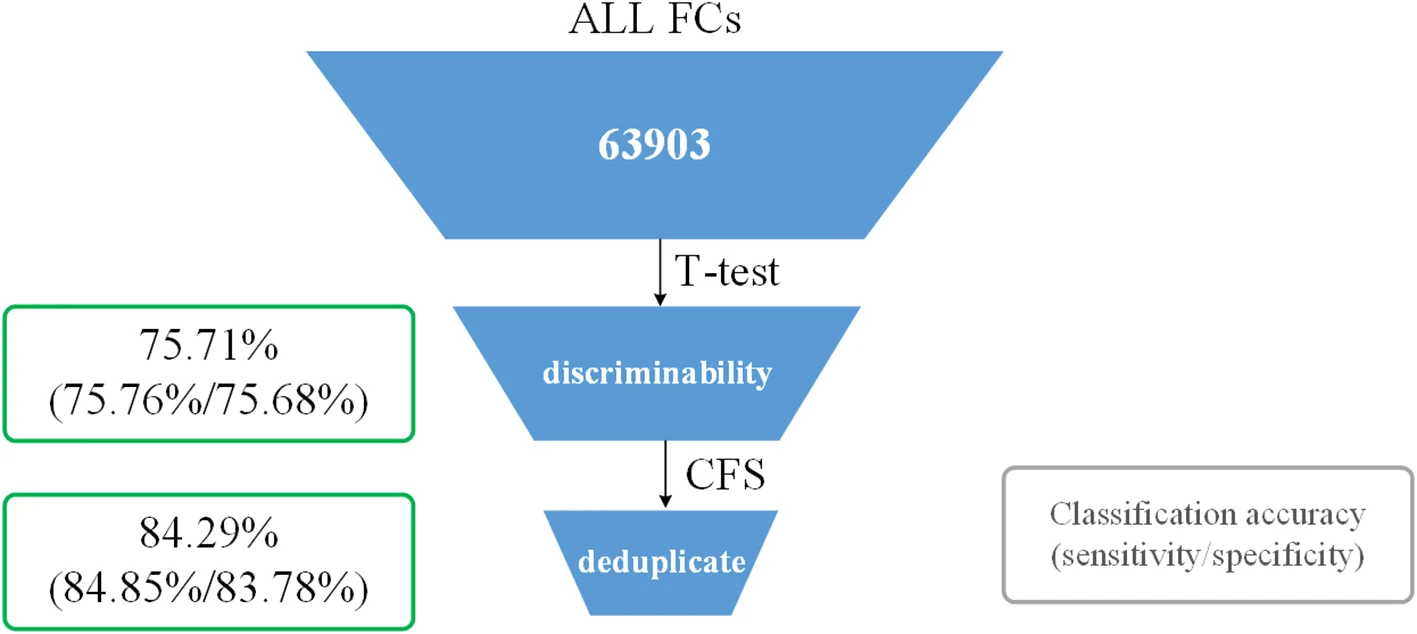
Leakage-free nested cross-validation framework for functional connectivity-based classification. Whole-brain functional connectivity features were derived from pairwise correlations among 358 DICCCOL landmarks, yielding 63, 903 FC features for each participant. Classification performance was evaluated using an outer leave-one-out cross-validation loop. In each outer iteration, one participant was held out as an independent test sample. All supervised procedures, including FDR-corrected group comparisons, correlation-based feature selection (CFS), feature standardization, and SVM hyperparameter optimization, were performed exclusively within the corresponding outer training data. Hyperparameter tuning was conducted using an inner stratified 5-fold cross-validation loop. The optimized pipeline was subsequently refitted using the complete outer training set and applied once to the held-out test participant. Final performance metrics, including the confusion matrix, receiver operating characteristic curve, area under the curve, and permutation-based significance level, were calculated from aggregated out-of-fold predictions. The number and identity of selected FCs were permitted to vary across outer-fold iterations, and feature-selection stability was quantified by selection frequency. FC, functional connectivity; CSF, Cerebrospinal Fluid.

Starting with 63, 903 pairwise connections, we first applied a t-test to retain features that were univariately significant. These features were then submitted to CFS for redundancy reduction and subset selection. Although some feature subsets did not reach significance individually, they could still demonstrate strong joint discriminative power (45). Due to the vast search space, we did not perform an exhaustive exploration of all feature–feature interactions. Nevertheless, by inputting the screened features into CFS (stage two), the algorithm effectively captured these combinatorial effects, thereby leveraging the discriminative information retained from the initial filtering stage. The number of features retained at each selection stage, along with the corresponding classification results is illustrated at **Figure 3**.

To better illustrate the advantages of our method, we reported not only the classification accuracy of the final features, but also the number of surviving connections and intermediate classification results at each stage of the feature selection process. By selecting the most relevant and discriminative functional connections, the classification accuracy was significantly improved, reaching 84.29%.

**Figure 4:**
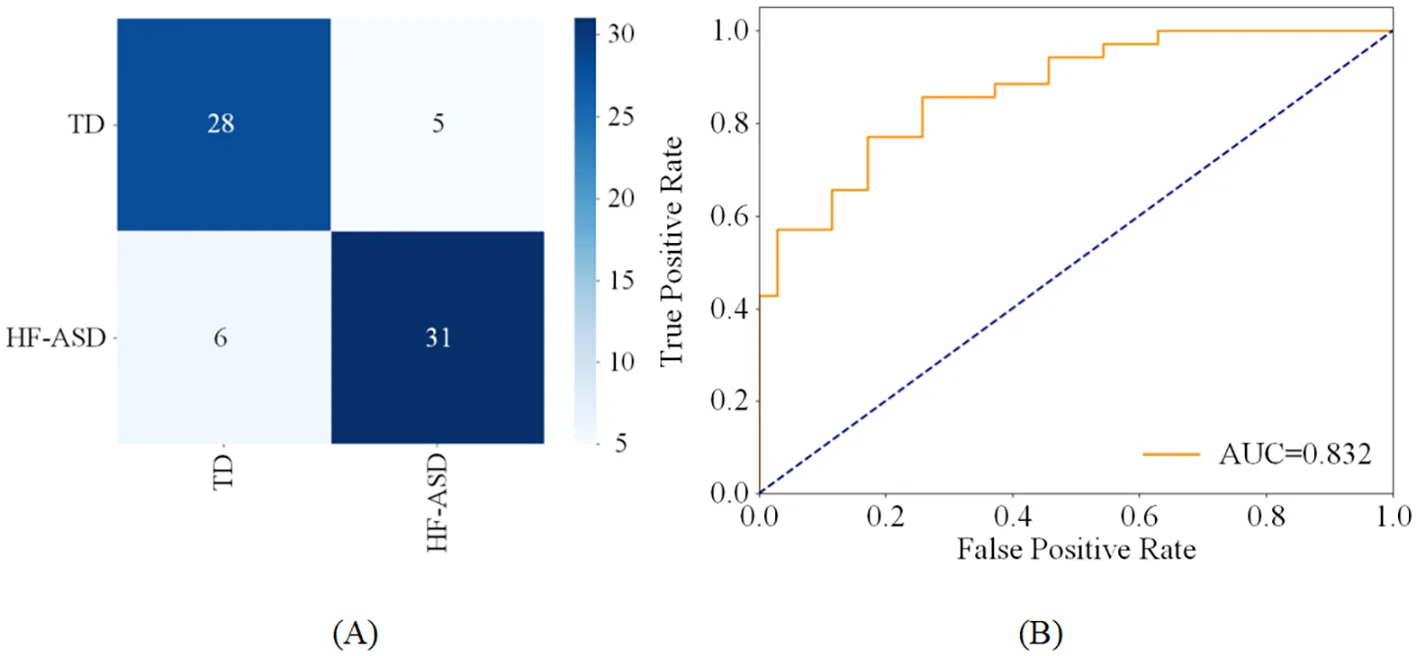
Classification performance of the DICCCOL-based functional connectivity model. **(A)** Confusion matrix derived from the aggregated out-of-fold predictions. Rows represent the true diagnostic labels, and columns represent the predicted labels. The model correctly classified 28 of 33 typically developing (TD) participants and 31 of 37 participants with high-functioning autism spectrum disorder (HF-ASD). **(B)** Receiver operating characteristic (ROC) curve of the classifier based on out-of-fold decision scores. The area under the ROC curve (AUC) was 0.832, indicating good discriminative performance for distinguishing HF-ASD from TD participants.

To further characterize the performance of the classification model, a confusion matrix and receiver operating characteristic (ROC) curve were generated based on the aggregated out-of-fold predictions. As shown in **Figure 4**, the model correctly classified 28 of 33 TD participants and 31 of 37 HF-ASD participants, yielding an overall classification accuracy of 84.29%. The sensitivity for identifying HF-ASD was 83.78%, whereas the specificity for identifying TD participants was 84.85%. In addition, the ROC analysis demonstrated an area under the curve (AUC) of 0.832, indicating good overall discriminative ability of the DICCCOL-based functional connectivity model for distinguishing children and adolescents with HF-ASD from TD controls. The following section analyzed the functional networks involved in the finally retained functional features (functional connectome).

### 3.3 Brain networks housing highly discriminative FCs

Following the two-stage feature selection process, we obtained 40 FCs with the strongest discriminative capacities between the two groups (achieving a classification accuracy of 91.43%), which we designated as the “connectome signature.” This section focused on the functional roles of the networks represented in these “connectome signatures.” Based on previous meta-analyses by Yuan et al (8). DICCCOLs have been successfully annotated with their corresponding functional roles (involved functional networks). Briefly, we registered the mean coordinates of each DICCCOL to a standard template space and performed a small-radius search to check for any reported functional task activations at those locations (15). If one or more activation reports were detected within the considered range, they were assigned to this DICCCOL as corresponding functional roles (46) (http://www.brainmap.org/sleuth/). For example, action included eight sub-functional networks such as execution, imagination, and inhibition. In total, we annotated 339 bifunctional networks with 55 sub-functional networks. Because the DICCCOLs involved in the “connectome signature” rarely originated from interoception networks, only the other four categories (47) were considered in this study. We quantitatively analyzed the composition of the obtained “connectome signature, “ with the details presented in Tables 3-2. The impaired brain networks in the HF-ASD group were primarily concentrated in the cognitive domain [executive control network, default mode network], perceptual domain (visual network), and affective domain (empathy network) (48).

**Figure 5:**
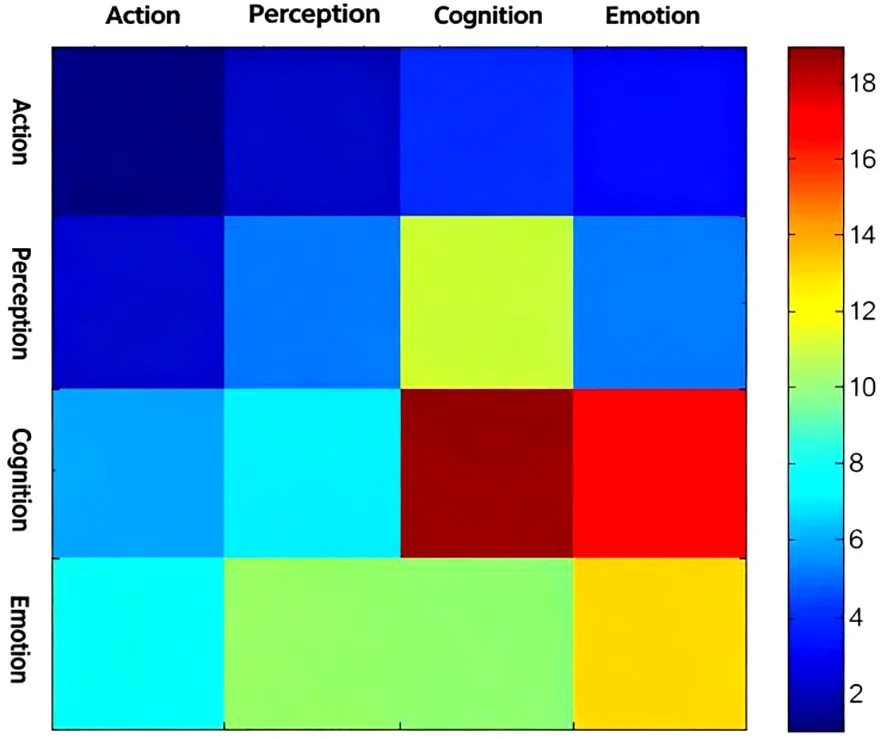
Representation of the functional relationship matrix corresponding to the selected highly discriminative “connectome signature.” Note: The heat map between Dense Individualized and Common Connectivity-based Cortical Landmark models and functional networks illustrates the frequency of functional connectivity involvement across all functional networks.

As shown in **Table 2**, the HF-ASD group exhibited increased strength in 30 highly discriminative FCs, and decreased strength in 10 FCs. These alterations included both intra-network and inter-network connections, with abnormal FCs generally more prevalent within networks than between networks (49). Because we functionally annotated each DICCCOL with its involved functional network, we could easily derive a Functional Relationship Matrix (FRM), shown in **Figure 5** to represent the “connectome signature.” For example, there may have existed an FCs between DICCCOL-A and DICCCOL-B, where DICCCOL-A belonged to the functional networks of action and perception, while DICCCOL-B belonged solely to perception. Thus, the functional connection between the two DICCCOLs contributed to both the action-perception and perception-perception relationships in the matrix. Action-perception is regarded as global interactions between different functional networks, while perception-perception is identified as intra-network behaviors (50). Even without directly identifying substantial connectivity consistency, interesting patterns were readily observed through the corresponding FRM (51); the number of inter-network FCs slightly outnumbered those of intra-network FCs within individual functional networks (52) as shown in **Figure 5**. Cognition-cognition, cognition-affective, and affective-affective interactions showed higher involvement (53). This pattern suggested that the FCs showing the greatest differential capacity primarily originated from within individual networks, rather than from global interactions between different networks.

**Table 2:** Summary of the “connectome signature”.

Involved connectivity		Involved DICCCOLs	
Total Increased Decreased	Total	Action Perception Cognition	Emotion
40 30 10	48	11/12.09% 22/24.18% 35/38.46%	23/25.27%

### 3.4 Associations between stable functional connectivity features and clinical measures

**Figure 6:**
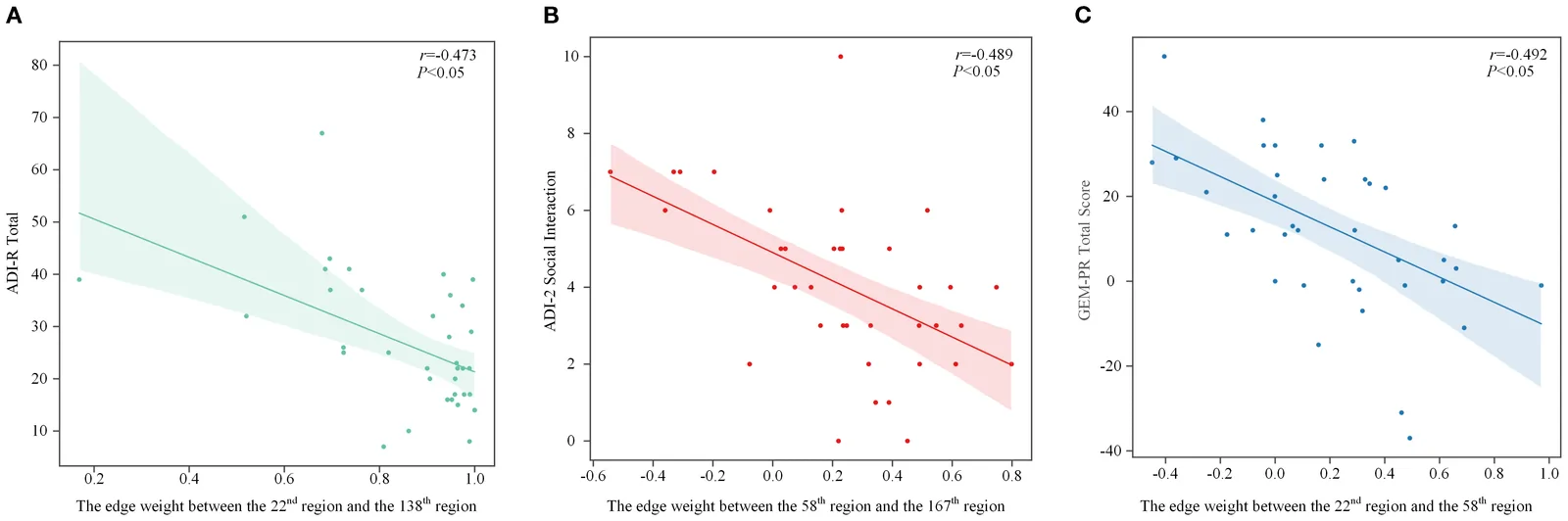
Representative associations between stable discriminative functional connectivity features and clinical measures in the HF-ASD group. **(A)** The functional connectivity strength between the 22nd and 138th DICCCOL regions was negatively correlated with the ADI-R total score (*r* = -0.473, *p* < 0.05). **(B)** The functional connectivity strength between the 58th and 167th DICCCOL regions was negatively correlated with the ADI-R Social Interaction score (*r* = -0.489, *p* < 0.05). **(C)** The functional connectivity strength between the 22nd and 58th DICCCOL regions was negatively correlated with the GEM-PR total score (*r* = -0.492, *p* < 0.05). Solid lines represent fitted linear regression lines, and shaded areas indicate 95% confidence intervals. ADI-R, Autism Diagnostic Interview-Revised; GEM-PR, Griffith Empathy Measure–Parent Rating; HF-ASD, high-functioning autism spectrum disorder.

Partial correlation analyses controlling for age, sex, full-scale IQ, and mean framewise displacement revealed significant inverse associations between several stable discriminative functional connectivity features and clinical measures in the HF-ASD group. the FDR-corrected clinical correlation analyses provided evidence that the functional connectivity strength between the 22nd and 138th DICCCOL regions was negatively correlated with the ADI-R total score (*r* = -0.473, *p* < 0.05). In addition, the connectivity strength between the 58th and 167th DICCCOL regions was negatively associated with the ADI-R Social Interaction score (*r* = -0.489, *p* < 0.05)). The connectivity strength between the 22nd and 58th DICCCOL regions was also inversely correlated with the GEM-PR total score (*r* = -0.492, *p* < 0.05) (**Figure 6**).

Collectively, these findings suggest that altered connectivity within the stable discriminative functional connectome may be associated with individual differences in autism-related symptom burden, social-interaction difficulties, and empathic functioning. Given the exploratory and cross-sectional nature of these analyses, the observed associations should be interpreted cautiously and require replication in larger independent samples.

## 4 Discussion

Based on DTI-derived ROIs (DICCCOLs), we constructed whole-brain functional connectomes and achieved highly discriminative “connectome signatures” within each group. This study demonstrated that children and adolescents with HF-ASD were distinguished from controls using our proposed “connectome signature, “ which exhibited both sensitivity and specificity. The results indicated that among the most discriminative FCs, interactions between different functional networks were significantly altered, while substantial changes were also observed in intra-network interactions, when compared with the control group.

The predominance of cognitive-cognitive, cognitive-affective, and affective-affective functional connections among the stable discriminative features may provide a neurobiological perspective on the social and emotional difficulties observed in HF-ASD. Cognitive-cognitive connectivity abnormalities, particularly those involving executive control- and default mode-related systems, may reflect disrupted integration of higher-order cognitive processes. Executive control systems are essential for attentional regulation, cognitive flexibility, response inhibition, and goal-directed behavior, whereas default mode-related processes contribute to self-referential processing, mentalizing, and the interpretation of social contexts. Altered interactions within and between these systems may therefore be associated with difficulties in flexible social adaptation, understanding others’ intentions, and integrating social information, all of which are commonly impaired in individuals with ASD.

Cognitive-affective connectivity abnormalities may indicate inefficient communication between systems supporting cognitive control and those involved in emotional appraisal and social-emotional processing. In typical social interactions, cognitive control mechanisms dynamically integrate contextual information with the emotional salience of facial expressions, vocal cues, and interpersonal behaviors. Disrupted cognitive-affective coupling may consequently contribute to atypical emotion recognition, reduced sensitivity to social reward, and difficulty generating context-appropriate emotional and behavioral responses in HF-ASD. In addition, affective-affective connectivity abnormalities may reflect altered coordination among neural systems involved in emotional salience detection, affective resonance, empathy, and emotion regulation. Such abnormalities may be relevant to the atypical social-emotional responsivity and impaired empathic functioning frequently reported in ASD. Collectively, these findings suggest that the identified functional connectome patterns may not only distinguish HF-ASD from TD participants, but may also reflect distributed network-level alterations related to social cognition and affective processing. Nevertheless, because this study used a cross-sectional design, these interpretations should be considered tentative and require confirmation in larger longitudinal and independent cohorts.

In previous whole-brain functional network analyses (54), including those conducted under normal control conditions or various brain states, the nodes of ROIs or neural networks were typically constructed by registering individual brains to a template space, where entire anatomically homogeneous regions were treated as a single functional unit (55). Subsequently, the brain rsfMRI signals were averaged within these relatively large brain regions (typically encompassing entire gyri or sulci) to obtain representative rsfMRI time series (56). This framework proved effective when the goal was to identify overall relationships between different anatomical regions; however, it had its limitations. As Liu Tianming and colleagues have reported (57), the results of whole-brain functional connectivity may be influenced by the variability of BOLD signals within template-defined anatomical regions. When considering a relatively large Brodmann area, such as the precentral gyrus, averaging the rsfMRI time courses of all voxels within this region may “smooth” some useful signals, thereby losing informational relevance in subsequent analyses. In contrast, the 358 DICCCOLs distributed across the entire cortical surface provided finer granularity, improved functional homogeneity, more precise functional localization, and automatically established cross-subject correspondences (15). Another advantage of the system was that it was defined on the cortical surface (GM), which enhanced the accuracy of extracting rsfMRI BOLD signals.

Importantly, the FDR-corrected clinical correlation analyses provided evidence that specific stable discriminative functional connections may be related to individual differences in symptom expression in HF-ASD. Higher connectivity strength between DICCCOL regions 22 and 138 was associated with lower ADI-R total scores, and stronger connectivity between regions 58 and 167 was associated with lower ADI-R Social Interaction scores. As higher ADI-R scores indicate greater autism-related symptom burden and social-interaction impairment, these inverse associations may suggest that relatively preserved connectivity within these specific pathways is associated with less severe core clinical manifestations. In contrast, the connectivity strength between regions 22 and 58 was negatively associated with the GEM-PR total score, indicating that stronger connectivity in this pathway was related to poorer empathic functioning. The divergent directions of these associations suggest that functional connectivity abnormalities in HF-ASD may not represent a uniform reduction or increase in network efficiency. Rather, distinct connectivity alterations may have differential functional significance, potentially reflecting a mixture of maladaptive dysconnectivity and compensatory network reorganization across cognitive, social, and affective systems. These findings are consistent with the marked clinical and neurobiological heterogeneity of ASD and further support the view that network-level abnormalities may contribute differentially to symptom severity, social functioning, and empathy-related processes. Nevertheless, because these analyses were exploratory and cross-sectional, the observed associations cannot establish causal relationships and should be interpreted cautiously. The complete results of the correlation analyses require replication in larger independent cohorts.

Recent multi-site studies have shown that resting-state fMRI-derived functional network features, including graph-theoretical measures and ICA-based spatial-temporal network representations, have potential for distinguishing individuals with ASD from typically developing controls (2–4). These studies provide important evidence that ASD-related functional network abnormalities may be detectable across heterogeneous datasets. However, direct comparison of classification performance across studies should be made cautiously. Reported model performance can be substantially influenced by differences in sample composition, age range, clinical heterogeneity, site number, MRI acquisition protocols, preprocessing procedures, feature construction strategies, classifier selection, and validation frameworks. In contrast to approaches based on conventional atlas-derived regions or ICA-defined components, the present study used DICCCOL landmarks to construct an individualized and structurally informed functional connectome with cross-subject correspondence. Our findings therefore suggest that DICCCOL-based functional connectivity may provide a complementary approach for identifying candidate network-level alterations associated with HF-ASD. Nevertheless, because the present study was conducted in a relatively modest single-center sample and did not include an independent multi-site external validation cohort, the generalizability and reproducibility of the proposed model require further evaluation in larger, multi-center, and independent datasets.

Several limitations should be acknowledged. First, this study relies on a relatively modest, single-center sample. Although the fully nested leave-one-out cross-validation framework maximizes data utilization and reduces the risk of information leakage, internal validation cannot replace external validation using an independent cohort. Moreover, the high dimensionality of whole-brain functional connectivity relative to the available sample size may increase the risk of model instability and produce optimistically biased performance estimates. Consequently, the current findings should be interpreted as preliminary evidence supporting the potential discriminative utility of DICCCOL-based functional connectivity patterns in HF-ASD, rather than as validation of a clinically established diagnostic biomarker. Second, the generalizability of the proposed model may be limited by inter-site differences in participant demographics, clinical profiles, MRI acquisition protocols, scanner hardware, and preprocessing pipelines. It is important to note that one of the advantages of the method was its ability to integrate and combine different datasets from various laboratories. Therefore, future studies should validate the model using larger, multi-center, independent datasets—ideally incorporating prospective external validation cohorts. These efforts should also assess the reproducibility of the selected functional connections, the classifier’s calibration and clinical utility, and its performance across diverse age ranges, symptom severities, and ASD subtypes. Additionally, implementing harmonization procedures to reduce site- and scanner-related variability should be prioritized in future multicenter investigations.

In the present study, we applied a structure-informed functional connectivity analysis based on DICCCOL landmarks to characterize whole-brain connectome alterations in children and adolescents with HF-ASD. Within a fully nested cross-validation framework, the two-stage feature-selection procedure identified stable discriminative functional connections that achieved an out-of-fold classification accuracy of 84.29%, with a sensitivity of 83.78%, a specificity of 84.85%, and an area under the receiver operating characteristic curve of 0.832. These findings indicate that DICCCOL-based functional connectivity features showed good discriminative potential for distinguishing HF-ASD participants from TD controls in the present sample. The identified stable functional connections included both hyperconnectivity and hypoconnectivity and were distributed across intra-network and inter-network interactions, particularly within cognitive-cognitive, cognitive-affective, and affective-affective systems. These distributed connectivity alterations may reflect abnormal functional integration and segregation across neural systems involved in social cognition, emotional processing, and empathic functioning. In addition, exploratory clinical correlation analyses showed that several stable functional connections were negatively associated with ADI-R total and Social Interaction scores, as well as GEM-PR total scores. These findings suggest that distinct connectivity alterations may be related to interindividual differences in autism-related symptom burden, social-interaction difficulties, and empathic functioning. Nevertheless, the observed clinical associations were exploratory and cross-sectional and therefore do not establish causal relationships between altered functional connectivity and clinical manifestations. Although the identified connectome patterns demonstrated discriminative and potentially clinically relevant value in the present sample, they should be considered candidate neuroimaging signatures rather than established diagnostic biomarkers. Their biological specificity, clinical relevance, reproducibility, and generalizability require rigorous validation in larger, longitudinal, independent, and multi-center cohorts.

## Data Availability

The raw data supporting the conclusions of this article will be made available by the authors, without undue reservation.

## Supplementary material

Supplementary Figure 1 Empirical null distribution generated from 5, 000 permutation tests. The histogram depicts the distribution of the test statistic obtained after randomly permuting group labels 5, 000 times while repeating the same analytical procedure used for the observed data. The red dashed line indicates the observed test statistic derived from the original labels. Green dotted lines indicate the two-sided critical values of the empirical null distribution. The observed statistic was located in the extreme right tail of the null distribution, supporting that the observed effect was unlikely to arise by chance.
