# Supplementary material for: Diagnostic classification of children and adolescents with high-functioning autism spectrum disorder based on brain functional network characteristics of the dense individualized and common connectivity-based cortical landmark model
Source: Front Psychiatry. 2026 Aug 18;17:1892660. doi: 10.3389/fpsyt.2026.1892660 (PMC13530007; doi:10.3389/fpsyt.2026.1892660)
Supplement: Supplementary file 1 [file Image1.pdf]

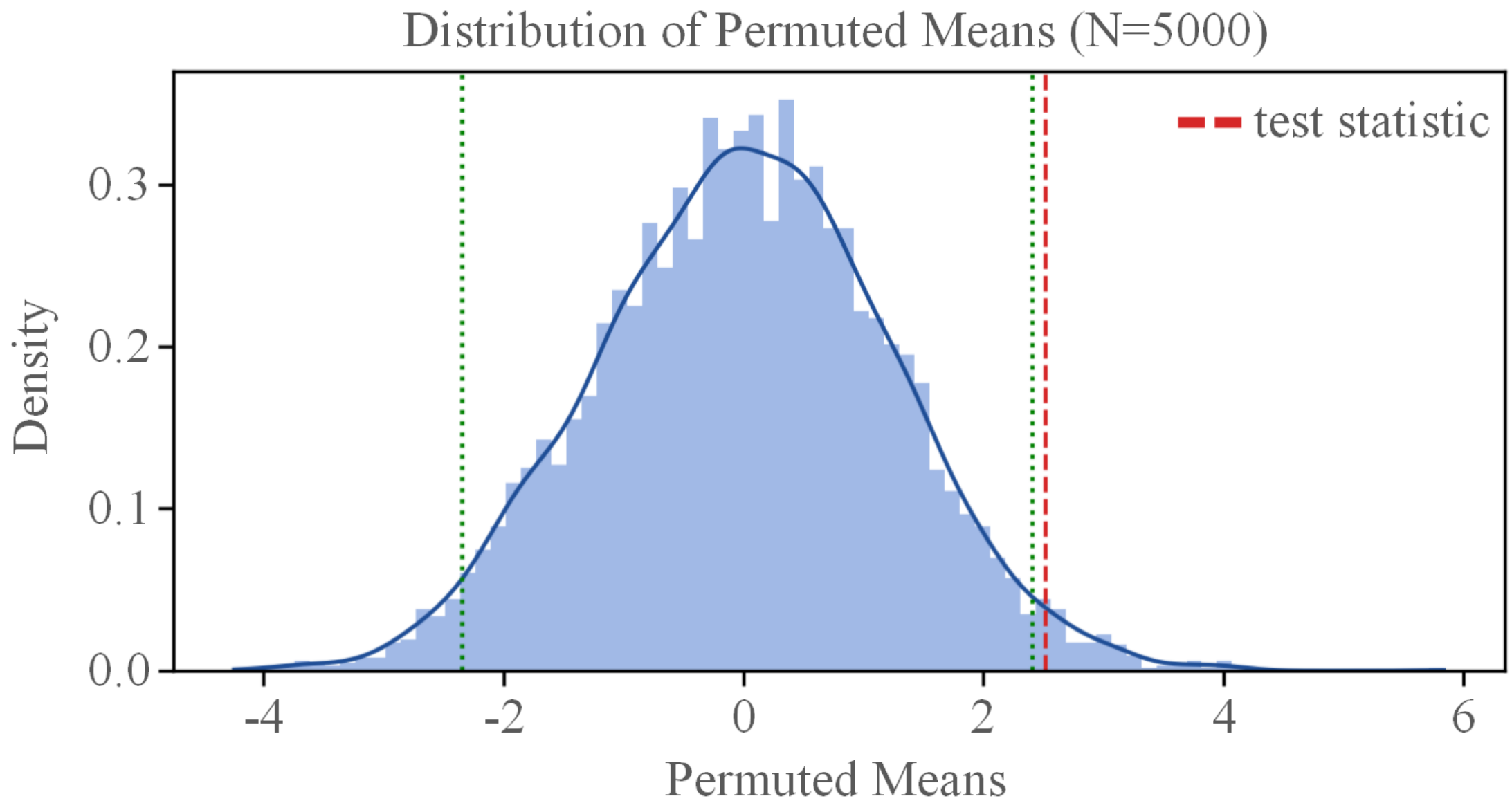

Supplementary Figure S1. Empirical null distribution generated from 5,000 permutation tests. The histogram depicts the distribution of the test statistic obtained after randomly permuting group labels 5,000 times while repeating the same analytical procedure used for the observed data. The red dashed line indicates the observed test statistic derived from the original labels. Green dotted lines indicate the two-sided critical values of the empirical null distribution. The observed statistic was located in the extreme right tail of the null distribution, supporting that the observed effect was unlikely to arise by chance.
